# An Electronic Registry for Improving the Quality of Antenatal Care in Rural Bangladesh (eRegMat): Protocol for a Cluster Randomized Controlled Trial

**DOI:** 10.2196/26918

**Published:** 2021-07-06

**Authors:** Anisur Rahman, Ingrid K Friberg, Akuba Dolphyne, Ingvild Fjeldheim, Fatema Khatun, Brian O'Donnell, Jesmin Pervin, Monjur Rahman, A M Qaiyum Rahman, U Tin Nu, Bidhan Krishna Sarker, Mahima Venkateswaran, J Frederik Frøen

**Affiliations:** 1 International Centre for Diarrhoeal Disease Research, Bangladesh (icddr, b) Dhaka Bangladesh; 2 Tacoma-Pierce County Health Department Tacoma, WA United States; 3 Global Health Cluster Division for Health Services Norwegian Institute of Public Health Oslo Norway; 4 Centre for Intervention Science in Maternal and Child Health (CISMAC) University of Bergen Bergen Norway

**Keywords:** quality of care, antenatal care, maternal and newborn health, eHealth, digital health interventions, eRegistries, health information systems, Bangladesh

## Abstract

**Background:**

Digital health interventions (DHIs) can alleviate several barriers to achieving better maternal and child health. The World Health Organization’s guideline recommendations for DHIs emphasize the need to integrate multiple DHIs for maximizing impact. The complex health system of Bangladesh provides a unique setting for evaluating and understanding the role of an electronic registry (eRegistry) for antenatal care, with multiple integrated DHIs for strengthening the health system as well as improving the quality and utilization of the public health care system.

**Objective:**

The aim of this study is to assess the effect of an eRegistry with DHIs compared with a simple digital data entry tool without DHIs in the community and frontline health facilities.

**Methods:**

The eRegMat is a cluster-randomized controlled trial conducted in the Matlab North and Matlab South subdistricts in the Chandpur district, Bangladesh, where health facilities are currently using the eRegistry for digital tracking of the health status of pregnant women longitudinally. The intervention arm received 3 superimposed data-driven DHIs: health worker clinical decision support, health worker feedback dashboards with action items, and targeted client communication to pregnant women. The primary outcomes are appropriate screening as well as management of hypertension during pregnancy and timely antenatal care attendance. The secondary outcomes include morbidity and mortality in the perinatal period as well as timely first antenatal care visit; successful referrals for anemia, diabetes, or hypertension during pregnancy; and facility delivery.

**Results:**

The eRegistry and DHIs were co-designed with end users between 2016 and 2018. The eRegistry was implemented in the study area in July 2018. Recruitment for the trial started in October 2018 and ended in June 2020, followed by an 8-month follow-up period to capture outcome data until February 2021. Trial results will be available for publication in June 2021.

**Conclusions:**

This trial allows the simultaneous assessment of multiple integrated DHIs for strengthening the health system and aims to provide evidence for its implementation. The study design and outcomes are geared toward informing the living review process of the guidelines for implementing DHIs.

**Trial Registration:**

ISRCTN Registry ISRCTN69491836; https://www.isrctn.com/ISRCTN69491836

**International Registered Report Identifier (IRRID):**

DERR1-10.2196/26918

## Introduction

### Background

The evidence-based interventions that can prevent maternal, newborn, and fetal deaths are well known [1], but the lack of access to timely and actionable data is a significant obstacle toward achieving better maternal and child health [2]. Much of the existing data for monitoring maternal and child health in low- and middle-income countries (LMICs) come from national surveys, such as the Demographic and Health Surveys [3] and the Multiple Indicator Cluster Surveys [4]. Health information systems in LMICs, which can provide routine and critical data, are often underutilized. In many settings, health information systems do not collect and manage data in ways that provide personalized good quality health care [5]. Other known issues such as the lack of awareness of guidelines, inadequate feedback to providers, and a poor coordination between the levels of health care have been shown to hamper effective data use [6].

Digital health interventions (DHIs) have the potential to address many of these issues. In 2017, the World Health Organization (WHO) published the Classification of DHIs, describing how digital tools can support health and health systems [7], whereas in 2019, their first recommendations for a select set of DHIs for health system strengthening were released [8]. Even for the DHIs included in the WHO guidelines, there is insufficient evidence to fully inform their optimal implementation in health systems. Evidence for the use of DHIs at a large scale in the real-world settings is crucial for policy makers to make decisions about system-wide implementations [9]. Every recent review of DHIs has unequivocally stated the need for more evidence of what works, and if and how such DHIs can be made scalable and sustainable [10,11]. DHIs are frequently tested in isolation or with weak study designs [8], making it difficult to ascertain their efficacy. The combinations of DHIs may be most effective in delivering measurable impacts on strengthening the health system. Many countries have transitioned to the use of digital health information systems. The enormous amount of data collected in such systems can be harnessed to drive several DHIs to strengthen the health systems, an opportunity often missed. Although evaluations of digital health information systems have focused on assessing timeliness and completeness of routine data, more comprehensive assessments of the outcomes of implementations of DHIs driven by data collected in health facilities are relatively rare.

An electronic registry (eRegistry) is a digital health information system comprising the longitudinal tracking of clients’ health status and services received, typically for maternal and child health [12]. In a health system with an eRegistry, health workers enter clinical data at the point of care into digital client health records. Data entry is typically set up as interactive checklists, which can minimize the documentation burden while enhancing data quality. The entered clinical data then drives several DHIs, for example, guideline-based clinical decision support for health workers [13] and targeted communication of personalized health information to clients [14]. An eRegistry can support additional DHIs such as feedback dashboards, stock notifications, routine health indicator data collection and management, and referral management. The specific set of DHIs included in an eRegistry, beyond the longitudinal tracking of clients’ health status, depends on the exact needs of a given implementation context.

Since 2018, an eRegistry has been implemented for use in Bangladesh in the Matlab North and Matlab South Upazilas, Chandpur district. Bangladesh has made significant progress in terms of maternal and child health. From 1990 to 2013, the Millennium Development Goal 4 was achieved (reduction of under-five mortality from 133 to 52 per 1000 live births), whereas maternal mortality declined significantly (from 550 to 196 per 100,000 live births) [15]. The primary health care system, where a large portion of maternal and child health services are provided, is a complex landscape of governmental, private, and nongovernmental organization (NGO) providers. The digital health atlas, an open-source platform for DHI implementations maintained by WHO, lists at least 12 registered implementations of DHIs in Bangladesh as of September 2020 [16].

The first cluster-randomized controlled trial of the effectiveness of an eRegistry with clinical decision support compared with paper records on improving the quality of antenatal care was conducted in the West Bank, Palestine [13]. The effect of an eRegistry with multiple DHIs such as health worker clinical decision support, feedback dashboards with action items, and targeted client communication, compared with a simple digital data entry tool with no DHIs, is yet to be evaluated.

### Objectives

The objective of this eRegistry trial (eRegMat) in Matlab, Bangladesh, is to assess the effect of an eRegistry with DHIs compared with a simple digital data entry tool without any DHIs in the community and frontline health facilities.

## Methods

### Trial Design

The eRegMat study is a two-arm cluster-randomized controlled trial. The unit of randomization is the primary care health facility, community clinics, and family welfare clinics. All pregnant women enrolled in the trial had their health information entered into the eRegistry. The intervention arm received 3 DHIs supported by the data in the eRegistry: (1) health worker clinical decision support, (2) feedback dashboards with action items, and (3) targeted client communication. The control arm received only a digital data entry tool without any additional DHIs.

### Study Setting

The public health system in two Upazilas (subdistricts)—Matlab South and Matlab North under Chandpur, Bangladesh—were included in the eRegMat trial. The public health system in Bangladesh is managed by the Ministry of Health and Family Welfare, where maternal and child health services are provided by two separate departments: the Directorate General of Family Planning and the Directorate General of Health Services. Under the Directorate General of Family Planning, family welfare visitors work at family welfare clinics located at the union level and family welfare assistants provide community outreach services. Under the Directorate General of Health Services, community health care providers offer maternal and child health as well as general health services from community clinics and health assistants who are tasked with visiting households and providing vaccination services to women and girls of reproductive age as well as young children through satellite clinics set up close to the community. A typical primary care health facility (community clinic or family welfare clinic) is permanently staffed by one clinical cadre (a community health care provider or a family welfare visitor). In addition, at the community clinics, the community health care provider is usually supported by a family welfare assistant or a health assistant. The community and facility workers cooperate to register pregnancies and encourage clients to seek reproductive, maternal, and child health services. In the existing paper-based health information system environment, there is no exchange of data or information between the different cadres of health workers or the different levels of the health system. The District Health Information Software 2 (DHIS2) [17] was used to generate aggregate reports of maternal and child health contacts in the study area.

In the study area, a substantial number of pregnant women access care at family welfare clinics and community clinics within the governmental health system. Antenatal care typically comprises the measurement of blood pressure, height, weight, fundal height; anemia assessment; the provision of vitamin and mineral supplementation; management of common illnesses; counseling; and referral, if needed. On the basis of these guidelines, pregnant women with complications are referred to a subdistrict (Upazila) hospital, where basic laboratory services as well as delivery services including cesarean sections are typically available. Some patients utilize tertiary care health facilities for delivery or when identified with complications during pregnancy. Women may also access private health care, where they are likely to receive ultrasound scans and laboratory tests. Most private hospitals perform cesarean sections. Several private and NGO-run facilities are also engaged in providing maternal and child health services in the study area.

### Eligibility Criteria

There were 2 levels of eligibility for this trial: (1) the cluster level, which refers to the randomized health facilities and their health workers and (2) the individual level, which refers to the women belonging to the clusters who provide informed consent.

#### Cluster Level

**Figure 1:**
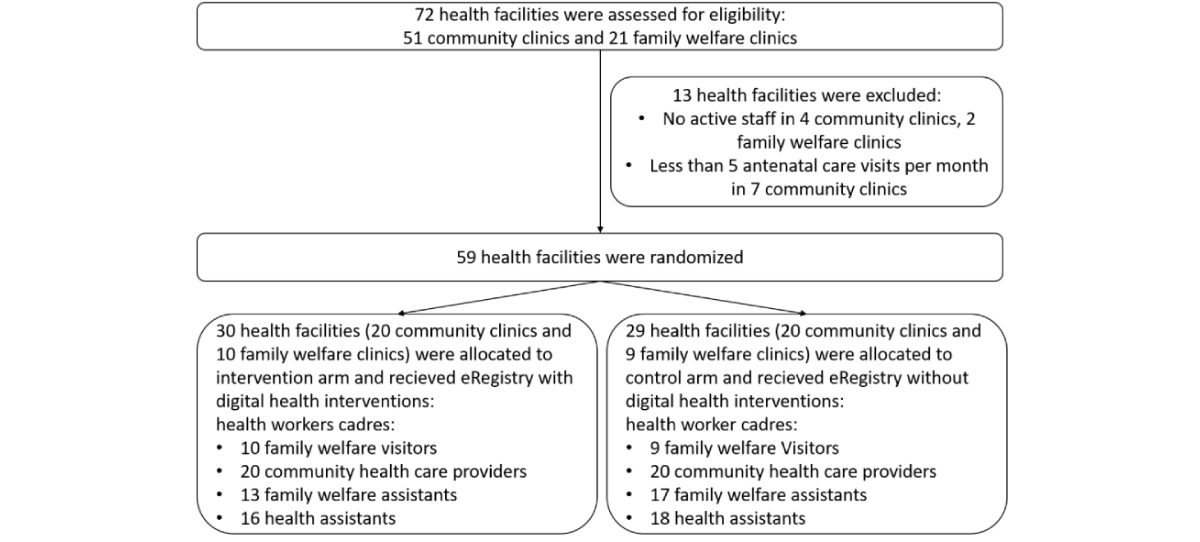
Flow diagram. eRegistry: electronic registry.

All government-run community clinics and family welfare clinics routinely providing antenatal care, delivery, or postnatal care services outside the referral health facility in the Matlab North and Matlab South Upazilas were eligible for the trial. Facilities were excluded if (1) there was no community health care provider or family welfare visitor to provide information on antenatal care visits in the previous month or (2) they reported fewer than five antenatal care visits in the previous month. Of the 72 health facilities in the study area, 59 (82%) were randomized (Figure 1). In total, 30 health facilities and 59 health workers were allocated to the intervention arm, whereas 29 health facilities and 64 health workers were allocated to the control arm (Figure 1).

#### Individual Level

Individual women with a confirmed pregnancy were registered, and their health data were entered into the eRegistry. Pregnancy identification and registration may be conducted by family welfare assistants or health assistants at the households or by community health care providers or family welfare visitors at the women’s first health facility encounter. All pregnancies identified by a health worker using the eRegistry should be entered into the system. Only women who consent to have a household visit after birth were included in the trial because the outcome data were derived from the household survey.

### Intervention

#### Overview

**Figure 2:**
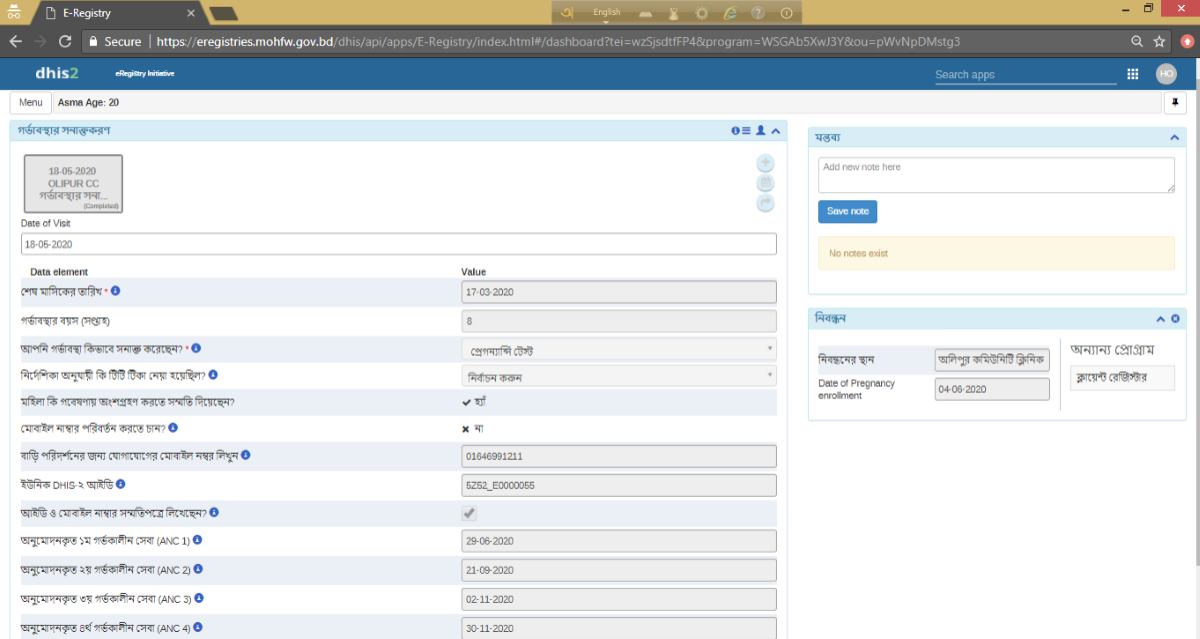
Screenshot of electronic registry interface for data entry in the study area—intervention and control clusters.

An eRegistry was implemented in the study area by the International Centre for Diarrhoeal Disease Research, Bangladesh (icddr, b), in collaboration with the Norwegian Institute of Public Health, the University of Oslo, and the Ministry of Health and Family Welfare in Bangladesh. Health workers in both the intervention and control arms document clinical information in the eRegistry’s digital client health records at the point of care. Health workers’ cadres under both the Directorate General of Health Services and Directorate General of Family Planning (Table 1) use the eRegistry to create client health records across otherwise disjointed silos of the health information system. The eRegistry is configured in the free and open-source DHIS2 tracker. Health workers in the community (family welfare assistants and health assistants) use the eRegistry on a customized Android Tracker app installed on handheld tablets, whereas facility-based users (community health care providers and family welfare visitors) access the eRegistry through a browser on Chromebook (Figure 2). All devices were equipped with SIM cards for prepaid mobile data access. To improve client identification for optimal data continuity in an environment with a few unique identifiers, a palm-based biometric software provided by Element biometrics [18] was made available to the users of the eRegistry. The Element biometrics app was installed on the same device used by the health workers to access the eRegistry. A unique identifier was generated for each client in the biometrics app. Health workers were trained to copy this identifier to the client’s records in the eRegistry. A unique identifier was also generated by the DHIS2 system during the registration of each pregnancy. A demo version of the eRegistry and DHIs is available upon request from the authors.

Paper-based documentation of pregnancies; register books with the number and dates of antenatal, postnatal, and delivery services; and public health reports were maintained along with the eRegistry in both intervention and control health facilities throughout the trial period. Table 1 provides a detailed comparison of the situation before eRegistry implementation in the governmental health system with the control and intervention arms of the trial.

**Table 1:** The paper-based health information system before the implementation of electronic registry, the electronic registry in the control arm, and the electronic registry with digital health interventions in the intervention arm.

Health information system feature		Cadres	Before the trial	Control arm	Intervention arm
**Health worker: all**					
	Access to own records	FWA^a^, HA^b^, CHCP^c^, FWV^d^	✓^e^	✓	✓
	Access to all records in the system	CHCP, FWV			✓
**Health worker: community**					
	Digital client health records	FWA, HA		✓	✓
	Client identification (biometric and palm-based) and registration	FWA, HA		✓	✓
	Feedback dashboards	FWA, HA			✓
**Health worker: facility**					
	Client identification (biometric and palm-based) and registration	CHCP^f^, FWV		✓	✓
	Digital client health records	CHCP^f^, FWV			✓
	Clinical decision support	CHCP^f^, FWV			✓
	Feedback dashboards	CHCP^f^, FWV			✓
**Health worker: all**					
	Access to own records	FWA, HA, CHCP, FWV	✓	✓	✓
	Access to all records in the system	CHCP, FWV			✓
**Clients**					
	Targeted Client Communication via SMS	Pregnant women			✓

^a^FWA: family welfare assistant.

^b^HA: health assistant.

^c^CHCP: community health care provider.

^d^FWV: family welfare visitor.

^e^Features of the eRegistry with and without digital health intervention available in the intervention and control arms.

^f^Community health care providers use the District Health Information Software 2 system for separate aggregate reporting, not linked to the electronic registry.

#### Control Arm

In the control arm of the trial, the eRegistry comprised only digital client health records (Table 1). The data points included in the client records were almost identical to the paper-based client records, and drop-down lists were added to some data points to optimize the use of the digital data entry tool. The eRegistry in the control arm was set up such that control users had no access to client records entered by other users. Apart from basic data validation warnings, data-driven DHIs were not available in the control arm (Table 1).

#### Intervention Arm

In the intervention arm, the eRegistry supported 3 data-driven DHIs linked to the digital client health records (Table 1) as follows: (1) for facility-based staff, health worker clinical decision support based on national guidelines to promote the correct management of health conditions identified in antenatal care; (2) for both field- and facility-based staff, feedback dashboards tracking the health worker’s progress toward pregnancy registration, antenatal care attendance and utilization, and clinical screening and management targets; and (3) for pregnant women, targeted client communication in the form of SMS text messages to address gaps in the timely attendance of antenatal care visits and facility delivery. Following the initial implementation of the eRegistry, the DHIs in the intervention arm were gradually introduced over the course of the trial; health worker clinical decision support was introduced in October 2018; targeted client communication via SMS and feedback dashboards were implemented in June 2019. Digital client health records were accessible across the cadres of health workers, facilities, and arms of the health system for a comprehensive longitudinal tracking of clients’ health status and services received (Table 1).

Guideline-based clinical decision support and feedback dashboards were developed in close collaboration with health workers. The decision support comprised referral and counseling reminders, medication alerts, and scheduling based on the recommended guidelines in the public health system. Feedback dashboards were customized for each cadre of health workers, with indicators of antenatal care attendance and screening and management of gestational diabetes, hypertension, and anemia. Targeted client communication included SMS text message reminders of routine antenatal care, referral facilitation SMS text messages for those at risk, and facility delivery reminders for those who are advised to have an institutional delivery. All DHIs were triggered by data entry into the eRegistry at the point of care by the health workers.

### Adherence

The Directorate General of Health Services in Bangladesh employs DHIS2 as their national health information system platform for aggregate data. A DHIS2 tracker app for individual-level data collection is available for one cadre of health workers, the community health care providers. The study team reinforced adherence to the use of the eRegistry in several ways. The study team members periodically attended monthly meetings with health workers. Technical support was provided for crosscutting issues around data entry, client identification, and verification to both the control and intervention arms throughout the trial. After the rollout of the eRegistry, all health workers were evaluated on key competencies in using the eRegistry and the Element biometrics app. Those identified with challenges in using digital tools were retrained. Field support staff from the study team also checked in periodically on such health workers to provide one-on-one support. The study team maintained a log of the most important issues that emerged with a possible direct impact on trial outcomes. Key indicators of adherence to the use of the eRegistry across intervention and control clusters will be presented along with the trial results.

### Concomitant Care

At the start of the trial, no other health information system studies or implementations were ongoing in the study area. Both the control and intervention arms of the trial received similar support in the use of the eRegistry for data entry. Intervention and control clusters offer the same clinical antenatal care services, and the national treatment and management guidelines set by the Ministry of Health and Family Welfare for maternal and child health apply to both. All health facilities in the Matlab North Upazila are a part of separate demand-side incentive programs to encourage women to attend antenatal and postnatal care and to have a facility delivery [19]. During the postpartum home visit, we will capture data that allow the estimation of the proportion of women who utilized these programs and subsequently account for possible effects of this on the trial outcomes.

### Outcomes

The outcomes were formulated based on their relevance to the health system context and were meant to capture the potential effects of the specific DHIs under assessment. Data for the primary (Table 2) and secondary outcomes (Table 3) will be derived from the eRegistry’s client health records and through postpartum home visit interviews.

**Table 2:** Primary outcomes, data sources, and definitions.

Primary outcome	Sources of data	Measurement sequence	Definition	Intervention subcomponents with most direct effect
Timely attendance at eligible ANC^a^ visits	Postpartum household survey	Registrations continuously at point of care Household visit after the completion of pregnancy	Proportion with timely attendance	Targeted Client Communication via SMS text message: ANC appointment reminders
Screening and management for hypertension in pregnancy	Case records from the eRegistry^b^	Registrations continuously at point of care	Proportion that has at least one ANC at a randomized health facility with: Blood pressure measurement at routine ANC visits, and Referred if blood pressure is high, or Referred if blood pressure is high outside of the routine ANC visit	Health worker clinical decision support Feedback dashboards with action items

^a^ANC: antenatal care.

^b^eRegistry: electronic registry.

**Table 3:** Secondary outcomes, data sources and definitions.

Secondary outcome	Sources of data	Measurement sequence	Definition	Intervention subcomponents with assumed direct effect
Timely first ANC^a^ visit	Case records from the eRegistry^b^ Postpartum household survey	Registrations continuously at point of care Household visit after the completion of pregnancy	Proportion of women that have an ANC visit at or before 16 weeks among those with a home pregnancy registration before 16 weeks	Targeted Client Communication via SMS text message: ANC appointment reminders
High-risk women successfully referred to a skilled provider for additional care	Case records from the eRegistry Postpartum household survey	Registrations continuously at point of care Household visit after the completion of pregnancy	Proportion of women that received additional care during pregnancy among those that are referred for severe anemia, or hypertension or diabetes during pregnancy	Health worker clinical decision support Targeted client communication via SMS: Referral reminders, high risk, facility delivery reminders
Facility delivery among those advised to deliver in a facility	Case records in the eRegistry Postpartum household survey	Registrations continuously at point of care Household visit after completion of pregnancy	Proportion of women that deliver in a facility among those that should delivery in a facility based on certain risk factors identified during ANC	Health worker clinical decision support Targeted client communication via SMS text message: high risk, facility delivery reminders
Births with severe morbidity or mortality among all women and among those with any risk identified	Case records in the eRegistry Postpartum household survey	Registrations continuously at point of care Household visit after completion of pregnancy	Proportion with a very preterm birth, very low birth weight, perinatal death, or hospitalization of the newborn for at least 7 days after birth among all women enrolled in the trial, and those with any identified risk during ANC	Health worker clinical decision support Targeted client communication via SMS text message: referral reminders
Severe postpartum anemia	Postpartum household survey	Household visit after the completion of pregnancy	Proportion of women with severe anemia in postpartum	Health worker clinical decision support Targeted client communication via SMS text message: referral reminders

^a^ANC: antenatal care.

^b^eRegistry: electronic registry.

### Sample Size

Sample size was calculated based on the 2 primary outcomes using the following assumptions: (1) a cluster size of 77 women and 140 antenatal contacts in the randomized health facilities for a 21-month enrollment period; (2) a 20% coverage of timely antenatal care visits and 12% of women screened and managed for hypertension in the control group, based on data from the 2014 Demographic and Health Survey; and (3) an a priori intracluster correlation coefficient of 0.1, commonly reported for such process outcomes [20]. The given sample size will have more than 80% power to detect a minimum clinically significant change from 20% to 33% for timely antenatal care visits and from 12% to 22% for hypertension screening and management. The sample size was calculated using Stata 16 (StataCorp).

### Recruitment

Both the intervention and control health facilities received the same number of initial training sessions. The initial training duration was decided based on the underlying skill level of the health workers and the complexity of the tasks within the eRegistry. In total, 276 health workers (102 family welfare assistants, 71 health assistants, 44 community health care providers, 20 family welfare visitors, 20 family planning inspectors, and 10 assistant health inspectors) were trained over 21 sessions in using the eRegistry. Retraining was provided to health workers in both the intervention and control arms at regular intervals of 3-6 months and over 36 sessions during the trial.

### Allocation

#### Randomization of Health Facilities

The unit of randomization was a health facility (community clinics or family welfare clinics) located in the study area, which provided antenatal, childbirth, and postpartum care. We performed stratified restricted randomization with a 1:1 ratio. The randomization was stratified based on the type of clinic (family welfare clinics and community clinics) and whether the facility was staffed with a care provider. The randomization within each stratum was then restricted based on (1) the clinic’s allocation as intervention or control within a preceding project in the study area for strengthening maternal and child health services; (2) the technological skills of the staff; (3) the monthly number of clients attending the clinic; and (4) the monthly number of antenatal care visits. To measure the technological skills of the staff, we created an index using information from a provider and a clinic survey conducted by the study team in preparation for the trial. The index included items such as health workers’ technological capabilities, such as the use of phones for SMS and internet, and their comfort using technology. Statisticians at the Centre for Intervention Science in Maternal and Child Health (CISMAC), University of Bergen, undertook randomization.

#### Individual Women Within the Trial

**Figure 3:**
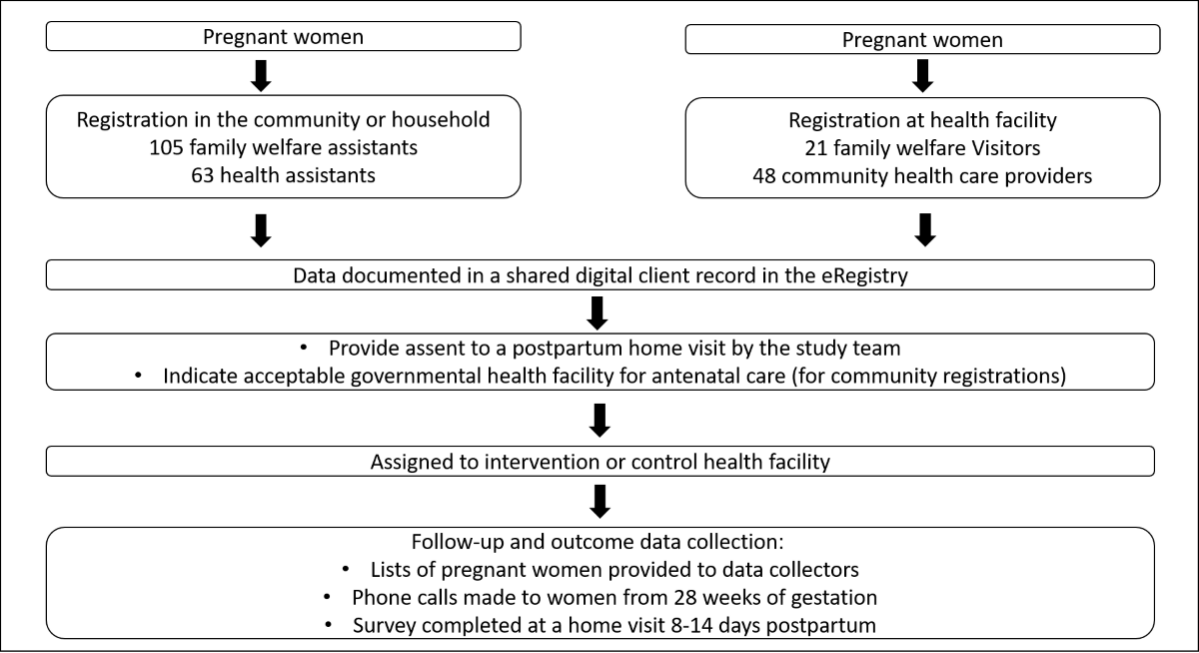
Identification and enrollment of individual pregnant women in the trial. eRegistry: electronic registry.

The family welfare assistant enrolled individual women in the trial at pregnancy identification and registration. At that time, women were required to provide informed consent to receive communication from the study team and a home visit 8-14 days after the birth of the child (Figure 2). Women were then asked about their preferred public health facility for antenatal care, which determined allocation to an intervention or control cluster (Figure 3). Women may also be first identified as pregnant at a health facility. At this contact, women were registered in the eRegistry and automatically assigned to an intervention or control cluster based on the allocation status of that health facility (Figure 3). After completion of the pregnancy, the data collection team conducted a postpartum home visit within 8-14 days of birth for collection of outcome data.

### Blinding

Due to the nature of the intervention, it was not possible to blind facility-based health workers to the allocation. Community-based health workers registering and allocating pregnant women were blinded to the allocation status of their preferred health facility. The data collectors conducting the postpartum home interviews were blinded to the allocation of individual women. Researchers performing the final analyses will be blinded to group allocation.

### Data Collection

#### Overview

The eRegistry used by health workers in the intervention and control arms will provide some of the data for outcome measurements. Independent trained project staff collected data regarding birth outcomes, antenatal care utilization, and referral care seeking from a subset of the population of registered pregnancies through a postpartum household survey. The postpartum household survey started in June 2019, 8 months after the commencement of recruitment. Data collectors were provided with a list of all pregnancies registered in the eRegistry that were beyond 28 weeks of gestation at a given point in time. The data collectors made biweekly phone calls to clients from 28 to 35 weeks, and weekly thereafter to ascertain whether the pregnancy was ongoing. Postpartum household visits for data collection were conducted within 8-14 days of childbirth. A paper-based structured questionnaire was used for data collection (Multimedia Appendix 1), focusing on the experiences of pregnancy and childbirth, health care interventions received, and health outcomes of the mother and the newborn. The infant’s weight was measured during the home visit; weight was measured in minimal clothing, and the weight of the clothes was subtracted from the total weight. Where possible, women’s finger-prick blood was used to measure hemoglobin. The questionnaires were checked for completeness and consistency by the data collectors’ supervisors, and women were interviewed again if needed. Data from the paper questionnaires are being made electronic, and consistency checks are performed by the study team after data entry.

#### Data Management

Data generated from this project are stored in the Government of Bangladesh’s server located at the office of the Directorate General of Health Services. For analysis purposes, an authorized data manager from the study team will extract predefined data from the server in a secure manner. Data from the postpartum household survey will be stored in locked cabinets in the icddr, b premises, with the personal identifiers removed and stored separately. Once these data have been entered digitally, the data manager will relink the eRegistry data with the postpartum survey data using the study ID. No other personal identifiers were available or were retained. The data will be saved in a password-protected computer. The data manager will share anonymous data sets with researchers for outcome analysis.

#### Statistical Methods

The primary analyses will be conducted as an intention-to-treat analysis, based on the allocation question rather than the actual site of antenatal care services, comparing the intervention arm with the control arm for all outcomes. Health worker characteristics such as gender, age, and education and health facility characteristics such as distance to referral unit, volume of pregnant women seen per month, digital competency, and internet connectivity will be presented. Pregnant women’s age, parity, socioeconomic status, and education will be presented as background characteristics. Primary analyses will be performed at the individual client level. Categorical variables will be analyzed using generalized linear models of the binomial family with a log link to estimate the relative risks and prevalence ratios. Random effects will be included to account for the inherent effect of clustering. Absolute and relative differences between the intervention and control groups with 95% CIs were calculated. Confounders will be adjusted for in the primary analysis only if there are important baseline imbalances (ie, when the effect measure changes by more than 5% when added to the model). Subgroup analyses will be performed based on categories defined as a priori, such as those related to demographic characteristics. We will present the equity analyses of the differential effectiveness across subgroups, including wealth quintiles, residence, educational levels, and employment status for each outcome. The imputation of missing data will be considered.

To understand the client base and their utilization of the health system, we will describe the client flow across providers, facilities, and arms of the trial. It is expected that some women will cross between the intervention and control clusters to receive health care services. Secondary analyses will be performed per protocol, where only the subset of women who remain in their assigned clinic type (ie, all visits are within the intervention group or control group) will be analyzed. We will perform a dose-response analysis (based on the proportion and number of visits to the intervention group) to assess whether there is a threshold or linear effect based on the increased utilization of intervention clusters. Finally, we will present population-level estimates of the impacts of the eRegistry if they were to be fully implemented in this community.

The study team will develop a code for analyses using dummy intervention or control allocation. A statistician not otherwise involved in the trial will conduct the final analyses using the actual allocations. All statistical analyses will be performed using statistical software such as Stata version 16 or later or RStudio version 1.2.1335 or later.

### Data Monitoring

The eRegMat trial is considered to be a health systems research where the intervention aims only to support existing care processes for care providers and does not constitute a medical intervention for individual subjects. Hence, a formal data safety monitoring board was not established.

#### Harms

As such, we do not anticipate any harm from the introduction of the intervention. The introduction of new technology may have resulted in reduced efficiencies during the initial months of utilization; however, we expect this to have resolved before the data inclusion period of this trial.

#### Audit

The CISMAC was responsible for trial monitoring. An initial monitoring visit was conducted in June 2018 to assess readiness before the start of recruitment. Midway, monitoring was conducted in November 2019, when recruitment rates, fidelity to the intervention, and progress of data collection were assessed.

### Ethics and Dissemination

#### Ethics and Consent to Participate

This study was reviewed and approved in Bangladesh by the Research Review Committee and Ethical Review Committee of icddr, b (Ref: PR-16054) and by the Regional Committee for Health Research Ethics–South East B Section (Ref: 2017/1028 C) in Norway. All health workers using the eRegistry and their supervisors at the Matlab North and Matlab South Upazilas were notified of their participation in the trial by the Ministry of Health and Family Welfare. No financial incentives were provided to the health facilities or care providers involved in this study; participation was considered mandatory by the central government. Financial support for participating in training sessions and meetings was conducted according to the government guidelines.

Informed consent would typically not be warranted for health systems research studies using anonymous health data, but three features in the implementation of this trial required additional ethical considerations. First, national guidelines recommend that family welfare assistants perform a urine pregnancy test for women who are uncertain of their pregnancy status. Because test kits were often unavailable in the health system, they were provided to health workers for the purpose of this trial. Women’s consent was required to obtain a urine sample for pregnancy testing. The sample never left the home, and the results were strictly confidential. If the woman declined to provide her sample, then the health worker privately counseled the woman of her options for testing. Second, the woman’s consent was required to receive targeted client communication via SMS text message. Women were informed about the purpose of the service, the content of SMS text message reminders, measures to ensure data confidentiality, and their ability to opt out of the service at any time. Providing a mobile phone number was assumed to imply consent. The option to stop receiving SMS text messages was available at every encounter of clients with their health workers. Phone numbers were available only to health workers who had access to the client’s health records. Third, all women enrolled in the eRegistry were asked to provide informed consent to undergo a postpartum home visit. This consent was for the purpose of being contacted by the study team during the latter part of their pregnancy and to allow the study team to conduct a visit 8-14 days postpartum. If women themselves notified the study team of the culmination of her pregnancy, a small incentive of 100 taka (approximately US $1) was given at the time of the home visit as a token of appreciation.

#### Confidentiality

The eRegistry data will be managed in accordance with the Ministry of Health and Family Welfare governance policies for health information systems. All data are stored securely on the national server under the custodianship of the Ministry.

#### Access to Data

The eRegistry data are owned by the Ministry of Health and Family Welfare in Bangladesh and subject to their regulations and legislation. Only one legally authorized person in the Bangladeshi Ministry of Health and Family Welfare will have access to individual-level data in the eRegistry. At no point will the researchers have access to identifiable data of any kind. The postpartum household questionnaire database will not be available outside the standard analysis protocols of the project, in accordance with the data access protocols of icddr, b.

#### Protocol Amendments

Changes in the secondary outcomes were written as amendments to the trial registration after being agreed upon in the study group. Amendments that amount to significant modifications to the study design have been reported to the CISMAC.

#### Collaborative Arrangements

The Norwegian Institute of Public Health owns the project and is ultimately responsible for the overall conduct; the institute has agreements with all the research partners detailing their roles and responsibilities. The icddr, b was responsible for the implementation and administration of the trial in Bangladesh, whereas the University of Oslo was responsible for the development of the eRegistry software in collaboration with other partners.

#### Dissemination Plan

The SPIRIT (Standard Protocol Items: Recommendations for Interventional Trials) guidelines were followed for writing this protocol (Multimedia Appendix 2). The trial publication will follow the CISMAC rules for authorship and publication and the Bangladesh Ministry of Health and Family Welfare regulations. We will publish the results in peer-reviewed open access journals and report them in accordance with the CONSORT (Consolidating Standards of Reporting Trials) guidelines.

### Funding

The Norwegian Research Council (project number 248073/H10; title: Strengthening the extension of Reproductive, Maternal, Newborn, and Child Health services in Bangladesh with an electronic health registry: A cluster randomized controlled trial) and the CISMAC, University of Bergen (project number 223269) funded this research. In addition to monetary inputs, CISMAC also provided intellectual input into the study design and monitoring of the trial.

## Results

Implementation of the eRegistry started in April 2018, after preliminary field testing (Table 4). Data documentation in the eRegistry started after the health workers completed a second round of training between July and December 2018. Data on all women enrolled between October 2018 and June 2020 (21 months) will be included in the analysis. The follow-up ended in February 2021, when the last enrolled woman received a postpartum household visit. Approximately 8000 pregnancies were registered in the eRegistry during the trial. The results of the trial are expected to be available in July 2021.

**Table 4:** Timeline of the eRegMat cluster-randomized controlled trial.

Implementation of the study		2018				2019					2020					2021
		Apr-May	Jul-Oct	Oct-Dec	Jan-Mar		Apr-Jun	Jul-Sep	Oct-Dec	Jan-Mar		Apr-Jun	Jul-Sep	Oct-Dec	Jan-Feb	
**Enrollment**																
	Trainings	✓^a^	✓				✓									
	Enrollment			✓	✓		✓	✓	✓							
**Intervention**																
	eRegistry^b^ (intervention and control)			✓	✓		✓	✓	✓	✓		✓				
**Assessment or follow-up**																
	Outcome data collection			✓	✓		✓	✓	✓	✓		✓	✓	✓	✓	

^a^Period during which the trial activity was conducted.

^b^eRegistry: electronic registry.

## Discussion

### Study Relevance

There is a global trend toward digital data collection in health systems in LMICs; however, the collected data are rarely used to support multiple DHIs. DHIs are shown to have relatively modest effects on their own, whereas a package of several simultaneously delivered DHIs is likely to be more effective and cost-effective. We designed a trial that compared two types of digital data collection systems: one where the longitudinally collected individual-level data in an eRegistry does not drive additional DHIs (control arm) and one where the data entered are used to generate three additional DHIs (intervention arm).

Antenatal care utilization in Bangladesh is suboptimal, with only 37% reported attendance of four or more visits [15]. In addition, gaps in antenatal care content and quality have been highlighted in studies conducted in other parts of Bangladesh [21]. The eRegistry’s data-driven DHIs aim to address antenatal care utilization (through targeted client communication via SMS text messages) as well as the quality of care (through health worker clinical decision support based on national guidelines), thereby addressing the two essential components of the effective coverage of antenatal care [22].

The transition of clinical documentation from paper to electronic data entry in digital client records may affect clinical practices and health care service delivery, even without any additional DHIs such as clinical decision support. In our trial, we wanted to evaluate the effects of data-driven DHIs per se, while controlling for the possible effects of the transition from paper to digital systems. Consequently, the control arm of the trial uses the eRegistry, albeit only as a digital data entry tool, with no superimposed DHIs.

The different government health workers in Bangladesh largely operate in silos, which limits the use of routinely reported data. The eRegistry spans multiple cadres of health workers and departments of the health system. The use of an eRegistry purposefully implemented for use by all cadres of health workers who typically meet an individual client in the public health system can provide more reliable data and improve the quality of health services. Health workforce capacity is an important component of the enabling environment for digital health [23]. In our study setting, except for the community health care providers, the other cadres were not skilled in using digital tools. Furthermore, health care workers do not receive in-service training in the use of digital tools. Low technology skills and lack of in-service training in the use of digital tools are common issues in several LMICs [24]. In addition to the results of the trial, we will report on the delivery of different components of the intervention, training needs, and implementation support over the course of the study. Such reporting would not only be in line with the general recommendations of reporting of trial of health systems and of DHIs [25] but also serve as useful guides for others planning studies of DHIs for health system strengthening in comparable health system settings.

### Strengths and Limitations

An important strength of the trial lies in the design and implementation of the intervention. We continuously engaged both health workers and clients to develop tailored DHIs. The eRegistry supports the creation of a single, individual-level health record across multiple cadres of health workers; few other initiatives have made efforts to implement such a system designed to provide continuity of data and care. The trial outcomes are meant to capture different aspects of the intervention, allowing for better interpretations of the effect while serving as another strength of the study.

This study had some limitations. Reduced fidelity to the use of the eRegistry as the primary documentation tool will adversely affect the DHIs driven by the entered data. The eRegistry is not set up to provide automated monthly public health reports. Public health reports are still largely paper-based, with one cadre of health workers using the DHIS2 system separately for aggregate reporting purposes. This continued use of paper-based documentation and aggregate reporting may lead to incomplete data entry into the eRegistry, thus affecting the optimal delivery of DHIs. The eRegistry is used only in public sector health clinics at the primary care level. As a result, the results of the trial are only indicative of care provision and utilization in the public sector. The health system landscape in our study area also comprises private and NGO-run health care providers, and clients are known to seek antenatal care from multiple providers. We will report on clients’ utilization of antenatal and delivery care from all health care providers, with the data collected during the postpartum household visit.

## Appendix

Multimedia Appendix 1 Postpartum household visit questionnaire.

Multimedia Appendix 2 SPIRIT (Standard Protocol Items: Recommendations for Interventional Trials) checklist.

## Abbreviations

CISMAC: Centre for Intervention Science in Maternal and Child Health
CONSORT: Consolidating Standards of Reporting Trials
DHI: digital health intervention
DHIS2: District Health Information Software 2
eRegistry: electronic registry
icddr, b: International Centre for Diarrhoeal Disease Research, Bangladesh
LMIC: low- and middle-income country
NGO: nongovernmental organization
SPIRIT: Standard Protocol Items: Recommendations for Interventional Trials
WHO: World Health Organization
