# Supplementary material for: An Electronic Registry for Improving the Quality of Antenatal Care in Rural Bangladesh (eRegMat): Protocol for a Cluster Randomized Controlled Trial
Source: JMIR Res Protoc. 2021 Jul 6;10(7):e26918. doi: 10.2196/26918 (PMC8292932; doi:10.2196/26918)
Supplement: Multimedia Appendix 1 [file resprot_v10i7e26918_app1.pdf]

eRegistry - Matlab Trial: Post-partum Home Visit Form

Date: |___|___| |___|___| |___|___|		Interviewers Code: |___|___|	
DHIS2 ID #			
**General information on participants in the eReg-Mat Trial**			
Time interview started: |___|___|:|___|___|			
Name of Woman:	Age: |___|___| Years		
Husband's name:	Age: |___|___| Years		
Address: Village: ___________ Union: ________	Upazila:	1=Matlab North	2=Matlab South |___|
LMP: |___|___| |___|___| |___|___|	Mobile No:		
Pregnancy outcome date: |___|___| |___|___| |___|___| (DD MM YY)			

No.	QUESTIONS & FILTERS	CODING RESPONSES			SKIP
SECTION 1: MOST RECENT PREGNANCY INFORMATION					
101	Pregnancy outcome	1=Alive	2=Stillbirth<br>3=Abortion/miscarriage/menstral regulation		201
102	Mode of delivery	Child 1	1= Normal delivery	2= C- section	|___|
		Child 2	1= Normal delivery	2= C- section	|___|
		Child 3	1= Normal delivery	2= C- section	|___|
103	What is the status of your child(ren) now?	Child 1	1= Alive	2= Dead	|___|
		Child 2	1= Alive	2= Dead	|___|
		Child 3	1= Alive	2= Dead	|___|
104	Date of neonatal death	Child 1	|___|___|-|___|___|-|___|___|		
		Child 2	|___|___|-|___|___|-|___|___|		
		Child 3	|___|___|-|___|___|-|___|___|		
105	Mother's current status	1= Alive		2= Dead	|___|
106	Date of maternal death	|___|___|-|___|___|-|___|___|			
SECTION 2: PREVIOUS PREGNANCY INFORMATION					
201	How many total pregnancies have you had, including live births, stillbirths, abortions, miscarriages, or those ended using menstrual regulation, including your most recent birth?	If none, record 00			|___|
202	How many total pregnancies have you had that ended in a live birth, including your most recent birth?	If none, record 00			|___|
203	How many total pregnancies have you had that ended in a stillbirth, including your most recent birth?	If none, record 00			|___|
204	How many living children do you have today, including your most recent birth?	If none, record 00			|___|
SECTION 3: RECENT PREGNANCY					
301	Now I would like to talk you about your <u>last</u> pregnancy. Did you receive check-up/antenatal care during this pregnancy?	1=Yes			|___|
		2=No			501
		9=Don't Remember			501
302	How many times did you seek antenatal care during your pregnancy?	|____|____| times<br>99= Unknown			
303	How long does it normally take to get from home to antenatal care?	|___|___| hours<br>|___|___| minutes			
304	Do you have your ANC card?	1=Yes	If yes, enter all dates below, and probe for more		|___|
		2=No	If no, use a calendar to estimate dates to enter below		
305	When was your first ANC visit?	Date:	|___|___| |___|___| |___|___|		
306	Whom did you see?	1= Doctors<br>2= Nurse/Midwife<br>3= Family Welfare Assistant<br>4= Family Welfare Visitor<br>5= Traditional Birth Attendant<br>6= Community Health Care Provider<br>7= Health Assistant<br>8= Village Doctor			|___|

		9= SACMO<br>10= Others (Specify)	
307	Where did you have that ANC visit?<br>Facility:___________________________	1=Home<br>2=H & FWC<br>3=Community Clinic<br>4=UHC<br>5=Other public facility<br>6=Other private facility<br>7= Satellite Clinic<br>8=Other (specify)	|___|
308	When was your second ANC visit?	Date: |___|___| |___|___| |___|___|	
309	Whom did you see?	1= Doctors<br>2= Nurse/Midwife<br>3= Family Welfare Assistant<br>4= Family Welfare Visitor<br>5= Traditional Birth Attendant<br>6= Community Health Care Provider<br>7= Health Assistant<br>8= Village Doctor<br>9= SACMO<br>10= Others (Specify)	|___|

310	Where did you have your ANC visit?<br>Facility:__________________________	1=Home<br>2=H & FWC<br>3=Community Clinic<br>4=UHC<br>5=Other public facility<br>6=Other private facility<br>7= Satellite Clinic<br>8=Other (specify)	|___|
311	When was your third ANC visit?	Date: |___|___| |___|___| |___|___|	
312	Whom did you see?	1= Doctors<br>2= Nurse/Midwife<br>3= Family Welfare Assistant<br>4= Family Welfare Visitor<br>5= Traditional Birth Attendant<br>6= Community Health Care Provider<br>7= Health Assistant<br>8= Village Doctor<br>9= SACMO<br>10= Others (Specify)	|___|
313	Where did you have your ANC visit?<br>Facility:__________________________	1=Home<br>2=H & FWC<br>3=Community Clinic<br>4=UHC<br>5=Other public facility<br>6=Other private facility<br>7= Satellite Clinic<br>8=Other (specify)	|___|
314	When was your fourth ANC visit?	Date: |___|___| |___|___| |___|___|	
315	Whom did you see?	1= Doctors<br>2= Nurse/Midwife<br>3= Family Welfare Assistant<br>4= Family Welfare Visitor<br>5= Traditional Birth Attendant<br>6= Community Health Care Provider<br>7= Health Assistant<br>8= Village Doctor<br>9= SACMO<br>10= Others (Specify)	|___|
316	Where did you have your ANC visit?<br>Facility:__________________________	1=Home<br>2=H & FWC<br>3=Community Clinic<br>4=UHC<br>5=Other public facility<br>6=Other private facility<br>7= Satellite Clinic<br>8=Other (specify)	|___|
317	When was your fifth ANC visit?	Date: |___|___| |___|___| |___|___|	
318	Whom did you see?	1= Doctors	|___|

2= Nurse/Midwife			
3= Family Welfare Assistant			
4= Family Welfare Visitor			
5= Traditional Birth Attendant			
6= Community Health Care Provider			
7= Health Assistant			
8= Village Doctor			
9= SACMO			
10= Others (Specify)			

319	Where did you have your ANC visit?<br>Facility:___________________________	1=Home	___
		2=H & FWC	
		3=Community Clinic	
		4=UHC	
		5=Other public facility	
		6=Other private facility	
		7= Satellite Clinic	
		8=Other (specify)	
320	When was your sixth ANC visit?	Date:	___ ___ / ___ ___ / ___ ___
321	Whom did you see?	1= Doctors	___
		2= Nurse/Midwife	
		3= Family Welfare Assistant	
		4= Family Welfare Visitor	
		5= Traditional Birth Attendant	
		6= Community Health Care Provider	
		7= Health Assistant	
		8= Village Doctor	
		9= SACMO	
		10= Others (Specify)	
322	Where did you have your ANC visit?<br>Facility:___________________________	1=Home	___
		2=H & FWC	
		3=Community Clinic	
		4=UHC	
		5=Other public facility	
		6=Other private facility	
		7= Satellite Clinic	
		8=Other (specify)	
If there are more than 6 ANC visits, complete an additional sheet of paper.			
Now I would like to talk to you about referrals from antenatal care during your last pregnancy.			
323	Were you told to seek any care or treatment from another source at any time during ANC?	1=Yes	___
		2=No →	401
324	Why were you refered for additional care?	1=Diabetes, or sugar	___
		2=Hypertension, pre-eclampsia, eclampsia	
		3=Anemia	
		4=Malpresentation of the baby	
		5=Other (specify)	
		99=Don't know	
325	Were you told to seek care or treatment from a specific source?	1=Yes	___
		2=No — If No →	327
326	Did you go to that location?	1=Yes	___
		2=No — If Yes →	328
327	Did you go anywhere for care?	1=Yes	___
		2=No — If No →	329
328	Where did you go?<br><br>RECORD ONLY ONE ANSWER- WHERE WOMAN OR FAMILY <u>FIRST</u> SOUGHT	1=Home	___
		2=H & FWC	
		3=Community Clinic	
		4=UHC	
		5=Other Public facility	

No.	Question	Response categories			Skip
	CARE/TREATMENT	6=Other private facility			
		7=Other (specify)			
329	Why did you <u>not go seek additional care?</u>	1=Woman Didn't Think Necessary			|___|
		2=Husband /Family Didn't Think Necessary			
	RECORD ALL RESPONSES	3=Facility too far			
		4=No Transport			
		5=No one available for child care			
		6=Could not afford it			
		7=Services are Poor Quality			
		8=Didn't Know Where To Go			
		9=No Time to Go			
		10=Other (Specify)			
		99=Don't Know			
SECTION 4: RECENT ANTENATAL CARE AND INCENTIVES					
401	Now I would like to ask you about your antenatal visits.		UNPROMPT	PROMPT	
	When you were pregnant with (NAME) (last pregnancy), what topics were you counseled about during at least one of your antenatal check-ups?	1=Breastfeeding	A	M	|___|
		2=Maternal Danger Signs	B	N	|___|
		3=Birth Planning	C	O	|___|
		4=Facility Delivery	D	P	|___|
		5=Nutrition in pregnancy	E	Q	|___|
	RECORD ALL RESPONSES	6=Post partum family planning	F	R	|___|
		7=Other (Specify)			
402	CHECK: ANY ANC AT CC OR FWC OR SC	1=YES ↓	2=NO →		406
403	During any ANC visit at a CC/FWC/SC did someone ever measure your blood pressure?	1=YES	2=NO	3=UNKNOWN	|___|
404	During any ANC visit at a CC/FWC/SC, did someone ever take a urine sample and test it? DO NOT INCLUDE PREGNANCY TESTS.	1=YES	2=NO	3=UNKNOWN	|___|
405	During any ANC visit at a CC/FWC/SC, did someone ever take a tiny blood sample from your fingertip?	1=YES	2=NO	3=UNKNOWN	|___|
SECTION 5: DELIVERY					
Now I would like to ask you some questions about the delivery of (NAME).					
501	Where did you give birth to (NAME)?	1=Home			|___|
		2=H & FWC			
		3=Community Clinic			
		4=UHC			
		5=Other public facility			
		6=Other private facility			
		7=Other (specify)			
502	Who delivered the baby?	1= Doctors			|___|
		2= Nurse/Midwife			
		3= Family Welfare Assistant			
		4= Family Welfare Visitor			
		5= Traditional Birth Attendant			
		6= Community Health Care Provider			

No.	Question	Coding categories			Skip / Code
		7= Health Assistant			
		8= Village Doctor			
		9= SACMO			
		10= Others (Specify)			
503	DELIVERED AT HOME?	1=YES	[illegible]O		510
					|___|
504	Were either of the following procedures performed at the time of delivery?	a. Received misoprostol	1=Yes	2=No	9=Don't know | |___|
		b. Injection	1=Yes	2=No	9=Don't know | |___|
505	When was misoprostol taken or inserted ?	1= Before the baby was born			|___|
		2= After the baby was born			
		3= Unknown			
506	What was the reason of using misoprostol?	1=Speed Up Labour			|___|
		2=Reduce Bleeding			
	RECORD ALL RESPONSES	3=Stop Convulsions			
		4=Other (Specify)			
		99=Don’t Know			
507	When was this injection administered?	1= Before the baby was born			|___|
		2= After the baby was born			
		3= Unknown			
508	What was the injection for?	1=Speed Up Labour			|___|
		2=Reduce Bleeding			
	RECORD ALL RESPONSES	3=Stop Convulsions			
		4=Other (Specify)			
		99=Don’t Know			
509	Who administered the injection?	1= Doctors			|___|
		2= Nurse/Midwifes			
		3= Family Welfare Assistant			
		4= Family Welfare Visitor			
		5= Traditional Birth Attendant			
		6= Community Health Care Provider			
		7= Health Assistant			
		8=Village Doctor			
		9=Paramedic			
		10= Others (Specify)			
Now I would like to ask you about any problems you experienced during your last pregnancy and delivery.					
510	Did you experience any of the following problems during the pregnancy, delivery, and /or up to 7 days after delivery of (NAME)?		**UNPROMPT**	**PROMPT**	
		A. EXCESSIVE BLEEDING	A	M	|___|
		B. HIGH FEVER	B	N	|___|
	RECORD ALL RESPONSES	C. BAD SMELLING VAGINAL DISCHARGE	C	O	|___|
		D. CONVULSION	D	P	|___|
		E. PROLONGED LABOR	E	Q	|___|
		F. RETAINED PLACENTA	F	R	|___|
SECTION 6: POSTPARTUM CARE FOR MOTHER					
Now I am going to ask you about the medical care you received for your self after the delivery of (NAME)					
601	DELIVERED AT HOME?	1=YES	[illegible]O		606

No.	Question	Response	Skip / Code
	Now, I would like to ask you some questions about your health after the time of delivery.		
602	After (NAME) was born, did you have a medical check-up?	1=Yes<br>2=No	|___| 2 → 606
603	Where did you receive this <u>first</u> check-up?<br><br>IF RESPONDED MORE THAN ONE PLACE, ASK ABOUT THE PLACE WHERE SHE WENT <u>FIRST</u> FOR CHECK-UP.<br><br>CIRCLE ONLY ONE RESPONSE	1=Home<br>2=H & FWC<br>3=Community Clinic<br>4=UHC<br>5=Other public facility<br>6=Other private facility<br>7=Other (specify)	|___|
604	How many days after the delivery did your first check-up take place?<br><br>RECORD '00' DAYS IF SAME DAY	Days |___|___| 99=Don't Know	|___|
605	Did you have this check-up because you were sick or was it a routine check-up?	1=Sick<br>2=Routine<br>3=Both<br>9=Don't Remember	
606	CHECK FIRST PAGE: BABY BORN DEAD ('2')?	1=YES → 801 2=NO	↓

SECTION 7: NEWBORN CARE UP TO 7 DAYS OF LIFE

No.	Question	Response	Skip / Code
	Now I am going to ask you about care of the newborn baby.		
701	Did (NAME) cry immediately after birth?	1=Yes<br>2=No<br>9=Don't Remember	1 → 704<br>|___|
702	Did (NAME) need help breathing or crying shortly after birth?	1=Yes<br>2=No<br>9=Don't Remember	|___| 2 → 704<br>9 → 704
703	What was done to help (NAME) cry or breath at the time of birth?<br><br>(DO NOT SUGGEST ANSWERS)<br><br>RECORD ALL RESPONSES	1=Nothing Mentioned<br>2=Dried The Baby<br>3=Rubbed Back<br>4=Rubbed The Feet<br>5=Mouth to Mouth Resuscitation<br>6=Heated the Cord<br>7=Slapped the Baby<br>8=Held the Baby Upside Down<br>9=Bag and Mask<br>10=Other (Specify)	|___|
704	After (NAME) was born, did any medical persons/ health worker check (NAME)'s health?	1=Yes<br>2=No<br>9=Don't Remember	|___| 2 → 708<br>9 → 708
705	How many days after the delivery did (NAME)'s first check-up take place?<br><br>RECORD '00' DAYS IS SAME DAY	DAYS AFTER DELIVERY |___|___| 99=DON'T KNOW/ REMEMBER	
706	Where did the <u>first</u> check-up take place?<br><br>Facility name:____________________________	1=Home<br>2=H & FWC<br>3=Community Clinic<br>4=UHC	|___|

		5=Other public facility	
		6=Other private facility	
		7=Other (specify)	
707	Was this check because (NAME) was sick or was it a routine check-up?	1=Sick	|___|
		2=Routine	
		3=Both	
		9=Don't Remember	

708	During the first week of life did (NAME) have any of the following problems?	Fever	1=Yes	2=No	9=Don't know	|___|
		Difficulty Breathing	1=Yes	2=No	9=Don't know	|___|
		Jaundice	1=Yes	2=No	9=Don't know	|___|
		Diarrhea	1=Yes	2=No	9=Don't know	|___|
		Umbilical Cord Infection	1=Yes	2=No	9=Don't know	|___|
		Convulsions	1=Yes	2=No	9=Don't know	|___|
		Feeding Problem	1=Yes	2=No	9=Don't know	|___|
		Others (specify)				

709	During the first week of life, was (NAME) ever admitted to the hospital for one of the previously mentioned problems?	1=Yes	|___|
		2=No →	713
		9=Don't Remember →	713
710	When was the baby first admitted to the hospital?	Date: |___|___| |___|___| |___|___|	
711	When was the baby first discharged from the hospital?	Date: |___|___| |___|___| |___|___|	

712	Was the baby admitted to the hospital more than 1 time?			day month yr	|___|
		1 = Yes	2nd	|___|___|-|___|___|-|___|___|	
			3rd	|___|___|-|___|___|-|___|___|	
			4th	|___|___|-|___|___|-|___|___|	
		2=No			
		9=Don't Remember			

713	At any time during your pregnancy/delivery/postpartum period did you receive any financial support or assistance from any source to encourage you to attend ANC?	1=Yes	|___|
		2=No	
		3= Don't Know	

SECTION 8:BIOLOGICAL

801	Baby's weight (kg)	|___|___|.|___|___|
802	Haemoglobin (g/dl) level of women	|___|___|.|___|

SECTION 9: SOCIO-ECOMIC STATUS

901	Women's education	**Types of institution**	**Years of education**
		1= School- Formal	|__|__|
		2= School-Nonformal	|__|__|
		3= Madrassah-Registered	|__|__|
		4= Madrassah-Unregistered	|__|__|
902	Husband's education	**Types of institution**	**Years of education**
		1= School- Formal	|__|__|
		2= School-Nonformal	|__|__|
		3= Madrassah-Registered	|__|__|

		4= Madrassah-Unregistered	|__|__|

Information on Land:

No.	Question	Decimal	Kani	Gonda	Kora
903	How much land do your household own?	**Decimal**	**Kani**	**Gonda**	**Kora**
	Homestead (including ponds & ditches)	|___|___|___|___|	|___|___|___|___|	|___|___|___|___|	|___|___|___|___|
	Agricultural land	|___|___|___|___|	|___|___|___|___|	|___|___|___|___|	|___|___|___|___|
			1 Kani= Decimal	1 Gonda= Decimal	1 Kora= Decimal

Source of Income:

No.	Question	Options	Code
904	a. During the last 12 months what were the sources of income of your household?	1. Agriculture (Own land)	|___|
		2. Agriculture (Share crops)	|___|
		3. Land/ pond mortage/ lease/ rent (in/out)	|___|
		4. Day labourer/ Rickshaw/ Van puller	|___|
		5. Skilled labourer (driver, carpenter,plumber, electrician)	|___|
		6. Selling/Catching Fish /Fish Farming	|___|
		7. Cattle/ Chicken/ Duck/ Poultry/Selling Milk	|___|
		8. Selling Handicrafts	|___|
	RECORD ALL RESPONSES	9. Tailoring Work	|___|
		10. Business (Small)	|___|
		11. Business (MEDIUM)	|___|
		12. Various Business (BIG)	|___|
		13. Paid Employment	|___|
	b. What was the source of income?	14. Pension	|___|
		15. Remittance (within country)	|___|
	i. |___|___|	16. Remittance (Outside country)	|___|
	ii. |___|___|	17. Food for work	|___|
	iii. |___|___| i. |___|___|	18. Old age/ destitute (allowances)/ VGD/ Other allowances (Freedom fighter), Chairman, Member	|___|
	v. |___|___|	19. House/ Shop rent	|___|
		20. Rent of Taxi, Tempo, Auto	|___|
		21. Boat/ Rickshaw/ van renting out	|___|
		22. Interest Business	|___|
		23. Journalist	|___|
		24. Others (specify)	|___|

Food security:

No.	Question	Yes	No	Code
905	Some households may not have food for all members three times every day for all times). Has your household been able to have food for all members for three times every day during the last one year?	1=Yes	2=No	|___|

Housing Facility:

No.	Question	Items	Code
906	a. How many rooms does your household have?		|___|___|
	b. Materials used for construction of the main dwelling	Items	
		1= Hemp/hay/bamboo, or other	|___|
		2= Mud brick, or C.I. sheet/wood	|___|
	(Write by observing)	3= Brick/cement	|___|

Commodities/ Assets

No.	Question	Item	Yes	No	Code
907	a. Does your household own the following commodities or assets?	Rickshaw/ Rickshaw van	1=Yes	2=No	|___|
		Auto rickshaw (CNG/ Battery/ Patrol)	1=Yes	2=No	|___|
		Power tiller (Cultivation)	1=Yes	2=No	|___|
		Pump (irrigation / water pump)	1=Yes	2=No	|___|
		Computer	1=Yes	2=No	|___|
		Solar panel	1=Yes	2=No	|___|

	RECORD ALL RESPONSES	Almirah/ Showcase	1=Yes	2=No	|___|
		Sofa set	1=Yes	2=No	|___|
		Television	1=Yes	2=No	|___|
		Mobile phone	1=Yes	2=No	|___|
		Bi-cycle	1=Yes	2=No	|___|
		Motor cycle	1=Yes	2=No	|___|
		Refrigerator	1=Yes	2=No	|___|
		Fan	1=Yes	2=No	|___|
	b. How many fans does your household own?			|___|___|	
	c. How many mobile phones does your household own?			|___|___|	
	d. Do you own/use your own phone?		1=Yes	2=No	|___|
Livestock					
908	Does your household own livestocks?		1=Yes	2=No	|___|
909	If yes, please mention type and number?		Type	Number	
		1	Duck and Chicken	|___|___|	
		2	Goat and Sheep	|___|___|	
		3	Cow and Buffalo	|___|___|	
Latrine					
910	Do you have any latrine?		1=Yes	2=No	|___|
911	What types of latrines are used by your household? (Write by observing)	1= Septic tank/ modern toilet			|___|
		2= Ring/Slab but waste not drained out			|___|
		3= Ring/Slab but waste drained out			|___|
		4= Concrete latrine but waste drained out			|___|
		5= Kancha (Earth) latrine (open)			|___|
		6= Open/Bush			|___|
		7= Others (Specify)			|___|
Drinking Water (with in 1 year)					
912	What are the main source (most commonly used) of drinking water?	1= Deep Tubewell			|___|
		2= Tubewell (Green)			|___|
		3= Tubewell (red)			|___|
		4= Tubewell not tested yet			|___|
		5= Supply water			|___|
		6= Rain water			|___|
		7= Pond			|___|
		8= River			|___|
		9= Ditch/ canal			|___|
		10= Others (specify)			|___|
Source of Light					
913	Main source of power/ electricity?	1= Kerosine Oil			|___|
		2= Electricity			|___|
		3= Solar Panel			|___|
		4= Generator			|___|
		5= Others (specify)			|___|
Fuel					
914	Type of fuel mainly used for cooking	1= Wood/Wood dust/Paddy Husk			|___|
		2= Leaves and straw			|___|
		3= Kerosine (stove)			|___|
		4= Gas (line)			|___|

5=Gas (Cylinder)	|___|
6= Biogas	|___|
7= Cowdung	|___|
8= Electric (Oven/ rice cooker/ micro-oven/ grill)	|___|
9= Others	|___|

End time: |__|__|:|__|__|

Edited By:	Code: |___|___|	Entry By:	Code: |___|___|
Date: |___|___| |___|___| |___|___|		Date: |___|___| |___|___| |___|___|	
